# The Effect of Changing Serum 25-Hydroxyvitamin D Concentrations on Metabolic Syndrome: A Longitudinal Analysis of Participants of a Preventive Health Program

**DOI:** 10.3390/nu7095338

**Published:** 2015-08-28

**Authors:** Truong-Minh Pham, John Paul Ekwaru, Solmaz Setayeshgar, Paul J. Veugelers

**Affiliations:** Population Health Intervention Research Unit, School of Public Health, University of Alberta 3-50 University Terrace, 8303-112 street NW, Edmonton, AB T6G 2T4, Canada; E-Mails: TruongMinh.Pham@ualberta.ca (T.-M.P.); ekwaru@ualberta.ca (J.P.E.); zohreh@ualberta.ca (S.S.)

**Keywords:** vitamin D, serum 25(OH)D, vitamin D supplementation, metabolic syndrome, longitudinal study

## Abstract

Several studies have shown that a poor vitamin D status may increase the risk of developing metabolic syndrome, which leaves the question whether improving one’s vitamin D status may reduce the risk for the syndrome. Here we investigate the effect of temporal changes in serum 25-hydroxyvitamin D (25(OH)D) concentrations on metabolic syndrome among Canadians enrolled in a preventive health program that promotes vitamin D supplementation. We accessed and analyzed data of 6682 volunteer participants with repeated observations on serum 25(OH)D concentrations and metabolic syndrome. We applied logistic regression to quantify the independent contribution of baseline serum 25(OH)D and temporal increases in serum 25(OH)D to the development of metabolic syndrome. In the first year in the program, participants, on average, increased their serum 25(OH)D concentrations by 37 nmol/L. We observed a statistical significant inverse relationship of increases in serum 25(OH)D with risk for metabolic syndrome. Relative to those without improvements, those who improved their serum 25(OH)D concentrations with less 25 nmol/L, 25 to 50 nmol/L, 50 to 75 nmol/L, and more 75 nmol/L had respectively 0.76, 0.64, 0.59, 0.56 times the risk for metabolic syndrome at follow up. These estimates were independent of the effect of baseline serum 25(OH)D concentrations on metabolic syndrome. Improvement of vitamin D status may help reduce the public health burden of metabolic syndrome, and potential subsequent health conditions including type 2 diabetes and cardiovascular disease.

## 1. Introduction

Metabolic syndrome is a constellation of conditions that include abdominal obesity, elevated blood pressure, elevated triglycerides, elevated fasting glucose, and reduced high-density lipoprotein (HDL) cholesterol [1,2]. This syndrome has been established as a risk factor for type 2 diabetes mellitus [3] and cardiovascular disease [3,4]. The prevalence of metabolic syndrome is increasing worldwide due to deteriorating lifestyles characterized by poor dietary habits and sedentary behaviors [5]. In Canada, a national study revealed that 20% of adults have metabolic syndrome, and that this estimate is expected to rise given the aging of the Canadian population [6,7]. Prevention of metabolic syndrome is an important public health objective as it will reduce of the future societal burden of diabetes and cardiovascular disease.

Vitamin D is known as the sunshine vitamin as it is synthesized in the skin by sunlight. Vitamin D can also be obtained orally through food and supplements. Vitamin D is metabolized to circulating 25-hydroxyvitamin D (25(OH)D) which is the established nutritional biomarker for vitamin D status [8,9]. The role of vitamin D for calcium homeostasis and bone health is established [9]. A role of vitamin D in the prevention of chronic diseases is suggested and in some cases documented [9,10,11,12,13]. Various cross-sectional studies, though not consistently, have reported inverse associations of higher serum 25(OH)D concentrations with lower prevalence of metabolic syndrome [14,15,16,17,18,19,20]. Ju *et al.* conducted a meta-analysis of these cross-sectional studies and estimated a pooled odds ratio of metabolic syndrome of 0.87 per 25 nmol/L increment in serum 25(OH)D concentration [20]. A nested case control study concluded the absence of a relationship between serum 25(OH)D concentrations and metabolic syndrome, whereas prospective analyses of cohort studies concluded the existence of this relationship [21,22,23]. These cohort studies revealed that baseline serum 25(OH)D concentrations determined the incidence of metabolic syndrome in the 3 and 5 years following baseline [21,22,23]. These observations were the basis of a call for intervention study [24].

For the present study, we accessed data collected as part of a preventive health program in which the supplementation of vitamin D is encouraged. We conducted secondary data analyses to confirm the findings from the above cohort studies and to investigated whether prospective increases in serum 25(OH)D concentrations would result in a reduction of the prevalence of metabolic syndrome.

## 2. Methods

### 2.1. Study Population

The Pure North S’Energy Foundation (PN) in Calgary, Alberta, Canada, is a not-for-profit organization that offers a preventive health program [25,26]. The program was launched in October 2007 with an initial focus on employees on remote sites in Northern Alberta, Canada, but was soon after opened to their spouses and to volunteer participants from across the province. In more recent years, the program broadened their focus to elderly and homeless populations. Participation was voluntary and free of charge. The program employs health professionals who provide informed lifestyle counseling. At enrollment, participants complete a lifestyle questionnaire, have a medical history and biometric measurements taken (height, weight, waist circumference, blood pressure) and have blood drawn for the assessment of serum 25(OH)D and various other biomarkers. The collected information serves the purpose of informing the health professionals as a basis for the lifestyle counseling. This counseling includes customized recommendations on diet, physical activity, sleep and stress management. Dietary supplementation is often encouraged and vitamin D supplementation in particular given Canada’s Northern latitude, limited sunlight and limited cutaneous synthesis of vitamin D. The program has used serum 25(OH)D concentrations of 120 nmol/L [27] as a basis for their recommendations for vitamin D supplementation, though their recommendations have changed over time and tailored to the specific preferences and needs of the participants. Follow up visits for health assessments and lifestyle counseling are scheduled annually. The primary objective of the PN program is lifestyle counseling and disease prevention rather than scientific research. However, the PN does make their collected data available, in anonymized form, to the University of Alberta to allow for secondary data analysis. For that purpose, participants signed and granted written informed consent to allow their relevant information to be used for secondary data analysis. The ethical approval for the use of the PN data for research and scientific reporting was granted by the Human Research Ethics Board of the University of Alberta.

The present analysis considered 7579 study participants who enrolled the program between 2007 and 2014, and who had at least one follow up visit in addition to their baseline assessment. We excluded 235 participants who fasted less than 8 h before blood samples were drawn. We also excluded 662 participants whose information was incomplete and metabolic syndrome could not be identified. As such, our analyses were conducted on a sample of 6682 participants including 3526 women and 3156 men.

### 2.2. Measurements for Serum 25(OH)D and Other Biomarkers

Participants were instructed to fast at least 8 h prior to having their blood samples drawn [26]. Serum 25(OH)D concentrations were measured by the Diasorin Liaison Direct using chemiluminescence immunoassay on an automated platform, and results were expressed as nanomoles per liter (nmol/L). Coefficient of variation (CV) of inter-assay for 25(OH)D measurements was 11%. Measurements for serum concentrations of triglycerides, HDL cholesterol, and fasting glucose were performed by automated Roche Cobas 8000 Modular Analyzer Series, and results were all expressed as millimoles per liter (mmol/L). The CV of inter-assay was 2% for triglycerides; 2% for HDL cholesterol; 1% for fasting glucose. Serum insulin concentrations were measured by the Abbott Architect Insulin on an automated immunoassay analyzer, and results were expressed as picomoles per liter (pmol/L) that were later divided by the constant of 6.945 to be converted into micro international units per milliliter (µIU/mL). The CV of inter-assay for insulin was 3%.

### 2.3. Metabolic Syndrome Determination

To identify metabolic syndrome we used the harmonized criteria [2] proposed and approved in 2009 by organizations such as the National Heart, Lung, and Blood Institute, the American Heart Association Science Advisory and Coordinating Committee, the International Diabetes Federation Task Force on Epidemiology and Prevention, among others. According to these criteria, metabolic syndrome is present if any three or more of the following conditions were met: (a) abdominal obesity, waist circumference ≥102 cm for men and ≥88 cm for women; (b) elevated blood pressure, ≥130/85 mmHg, or self-report of antihypertensive medications taken; (c) elevated serum triglycerides concentration, ≥1.7 mmol/L, or self-report of treatment for lipid profile; (d) elevated serum fasting glucose concentration, ≥5.6 mmol/L, or a diabetes status identified by either a self-reported history of diabetes, or medication taken for diabetes; (e) decreased serum HDL cholesterol concentration, <1.03 mmol/L for men and <1.30 mmol/L for women.

### 2.4. Assessment for Other Variables

We obtained the following information on confounding variables from questionnaires completed by the participants: age, sex, season when blood samples were drawn, smoking habits (categorized as never smoker, past smoker, and current smoker), alcohol consumption (non-drinker, drinker) and physical activity. Missing values for smoking, alcohol use, and physical activity were considered as missing categories in the regression analyses. Height and weight were measured at each visit and body mass index (BMI) was computed as weight in kilograms divided by square of height in meters (kg/m^2^). We further calculated the homeostatic model assessment for insulin resistance (HOMA-IR) according to the following equation [28]: HOMA-IR = (FI × FG)/22.5 in which FI denotes fasting serum insulin concentrations (in µIU/mL) and FG fasting serum glucose concentrations (in mmol/L).

### 2.5. Statistical Analyses

Baseline serum 25(OH)D concentrations were considered as the following categories: “less than 50”, “50 to less than 75”, “75 to less than 100”, “100 to less than 125”, and “125 or more” nmol/L. In the present study we considered the group with serum levels of “less than 50 nmol/L” as the referent group since 50 nmol/L is the minimum desirable level recommended by the Institute of Medicine [29]. Changes in serum 25(OH)D concentrations were calculated by subtracting the baseline concentration from the follow up concentrations, and were annualized by dividing it by the time (in years) between the baseline and follow up visit. These annualized changes were considered as the following categories: “No improvement” (for values of 0 or less), “Increase of less than 25”, “Increase of 25 to less than 50”, “Increase of 50 to less than 75”, and “Increase of 75 or more” nmol/L.

As descriptive statistics we present baseline and follow up values for metabolic syndrome and its five components, serum 25(OH)D concentrations, and potential confounders. As various participants had more than one follow up visit, we used the most recent follow up visit when presenting the descriptive statistics. However, we used all follow up information in all subsequent analyses. We applied logistic regression to quantify the relation of baseline 25(OH)D and changes in 25(OH)D with the presence of metabolic syndrome at follow up. These regression analyses were adjusted for sex, age, season when blood samples were drawn, smoking status, drinking status, physical activity level at baseline, physical activity changes during follow up, and the presence of metabolic syndrome at baseline. These logistic regression analyses were executed using generalized estimating equations methods to accommodate the fact that some participants had more than one follow up visit. In subsequent analyses we further adjusted these analyses for body mass index and insulin sensitivity at baseline. The above analyses were conducted for the population of 6682 participants who had at least one follow up visit, as well as for the subgroups of participants that had no metabolic syndrome at baseline, and had no metabolic syndrome and were not taking vitamin D supplementation at baseline. These latter “clean sample analyses” may be particularly relevant to primary prevention of metabolic syndrome. All statistical analyses were performed using SAS 9.4 (SAS Institute Inc., Cary, NC, USA). All statistical tests were two-sided and the level of statistical significance was at 0.05.

## 3. Results

**Figure 1:**
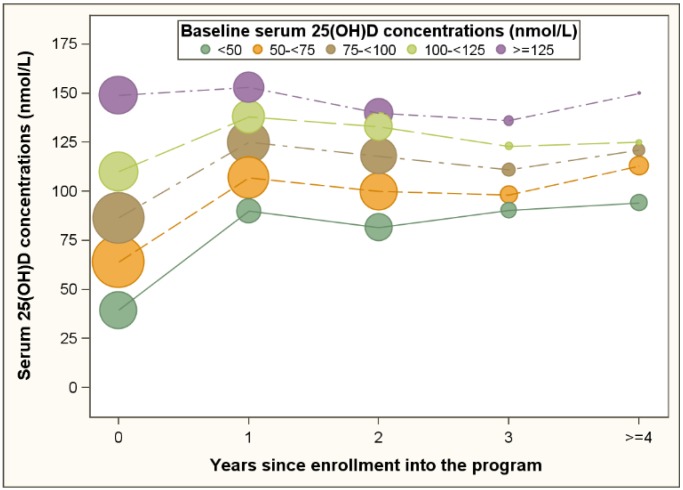
Serum 25(OH)D concentrations of program participants during follow up and grouped by baseline 25(OH)D concentrations. 25(OH)D, 25-hydroxyvitamin D; the sizes of the bubbles are proportional to the numbers of participants.

Of the 6682 participants, 4358 had one follow up visit, 1708 had 2 follow up visits, and 616 had 3 or more follow up visits. The median time between baseline and follow up was 1.1 year. Table 1 presents characteristics at baseline and the follow up visit. The prevalence of metabolic syndrome increased from 18% to 21%. The prevalence of elevated waist circumference and elevated blood pressure decreased whereas the prevalence of elevated triglycerides, elevated fasting glucose and reduced HDL cholesterol increased (Table 1). Average serum 25(OH)D concentrations increased from 89 nmol/L at baseline to 121 nmol/L at the follow up visit (Table 1). Figure 1 depicts the median serum 25(OH)D concentrations during follow up for five subgroups according to baseline concentrations. With the exception of the subgroup with baseline serum 25(OH)D concentrations above 125 nmol/L, 25(OH)D concentrations increased substantially in the first year of program enrollment and leveled off in subsequent years. For 1980 (19.8%) of the follow up visits no increases relative to the baseline 25(OH)D concentration were observed. Increases of less than 25 nmol/L, 25 to less than 50 nmol/L, 50 to less than 75 nmol/L, and 75 nmol/L or more were observed for 30.8%, 19.9%, 10.7% and 18.8% of follow up visits, respectively (Table 2).

**Table 1:** Baseline and follow up characteristics of 6682 study participants.

	Baseline	Follow Up
**Serum 25(OH)D, nmol/L**		
Mean (SD)	89 (42)	121 (46)
Median (IQR)	82 (61–108)	115 (89–147)
**Metabolic syndrome, *n* (%)**	1172 (18)	1393 (21)
**Metabolic syndrome components, *n* (%)**		
Elevated waist circumference	2469 (37)	2433 (36)
Elevated blood pressure	2442 (37)	2367 (35)
Elevated triglycerides	1290 (19)	1460 (22)
Elevated fasting glucose	1149 (17)	1417 (21)
Reduced HDL-cholesterol	1160 (17)	1482 (22)
**Women, *n* (%)**	3526 (53)	3526 (53)
**Age, mean (SD)**	51 (15)	52 (15)
**Season, *n* (%)**		
Summer	1235 (19)	954 (14)
Fall	1015 (15)	966 (15)
Winter	2665 (40)	2753 (41)
Spring	1767 (26)	2009 (30)
**Tobacco smoking status, *n* (%) ^a^**		
Never smoker	2680 (56)	1964 (57)
Quit smoker	1505 (31)	1021 (30)
Current smoker	605 (13)	458 (13)
Missing	1892	3239
**Alcohol drinking status, *n* (%) ^a^**		
Non-drinker	1744 (38)	1891 (40)
Drinker	2850 (62)	2857 (60)
Missing	2088	1934
**Physical activity, *n* (%) ^a^**		
Low	1932 (40)	1806 (37)
Moderate	1471 (31)	1549 (31)
High	1418 (29)	1586 (32)
Missing	1861	1741

25(OH)D, 25-hydroxyvitamin D; HDL-cholesterol, high density lipoprotein cholesterol; nmol/L, nanomoles per litter; SD, standard deviation; IQR, interquartile range. ^a^ Percentage for these variables do not include missing observations.

Table 2 presents the results of the univariate and multivariable analyses of the risk of metabolic syndrome at follow up. The results of the multivariable analyses differed from those of the univariate model suggesting the necessity of adjustment for all variables. Relative to participants with baseline serum 25(OH)D concentrations of less than 50 nmol/L, multivariable analysis showed that those with concentrations of 50 to less than 75, 75 to less than 100, 100 to less than 125, and 125 nmol/L or more at baseline were respectively 0.81, 0.51, 0.40 and 0.27 times less likely to have metabolic syndrome at follow up. Repeating the analyses presented in Table 2 but further adjusted for body mass index at baseline revealed that relative to participants with baseline serum 25(OH)D concentrations of less than 50 nmol/L, those with concentrations of 50 to less than 75, 75 to less than 100, 100 to less than 125, and 125 nmol/L or more at baseline were respectively 0.82 (95% CI: 0.65–1.02), 0.60 (0.47–0.76), 0.53 (0.39–0.70), and 0.41 (0.30–0.55) times less likely to have metabolic syndrome at follow up. When adjusting for HOMA-IR at baseline, those with serum 25(OH)D concentrations of 50 to less than 75, 75 to less than 100, 100 to less than 125, and 125 nmol/L or more at baseline were respectively 0.84 (95% CI: 0.67–1.05), 0.55 (0.44–0.70), 0.44 (0.33–0.59), 0.32 (0.24–0.43) times less likely to have metabolic syndrome relative to those with concentrations of less than 50 nmol/L.

**Table 2:** Risk for metabolic syndrome at follow up among 6682 study participants with a total of 10,019 follow up visits.

		Univariate Analysis		Multivariable Analysis ^a^	
	# visits	OR (95% CI)	*p*	OR (95% CI)	*p*
**Baseline 25(OHD), nmol/L**					
<50	1556	Reference		Reference	
50–<75	2775	0.88 (0.74–1.05)	0.17	0.81 (0.65–1.00)	0.06
75–<100	2535	0.54 (0.45–0.65)	<0.01	0.51 (0.40–0.64)	<0.01
100–<125	1609	0.39 (0.31–0.49)	<0.01	0.40 (0.30–0.53)	<0.01
≥125	1544	0.32 (0.25–0.40)	<0.01	0.27 (0.20–0.36)	<0.01
**Changes in 25(OH)D during follow up compared with baseline, nmol/L**					
No improvement	1980	Reference		Reference	
Increase of <25	3087	1.27 (1.08–1.50)	<0.01	0.76 (0.62–0.93)	0.01
Increase of 25–<50	1994	1.22 (1.03–1.45)	0.02	0.64 (0.52–0.80)	<0.01
Increase of 50–<75	1069	1.20 (0.98–1.47)	0.06	0.59 (0.46–0.77)	<0.01
Increase of ≥75	1889	1.23 (1.03–1.46)	0.02	0.56 (0.44–0.70)	<0.01
**Baseline metabolic syndrome**	10019	19.7 (17.0–22.8)	<0.01	16.7 (14.3–19.4)	<0.01
**Men *vs.* women**	10019	1.34 (1.19–1.51)	<0.01	1.24 (1.07–1.43)	<0.01
**Age at baseline**	10019	1.02 (1.02–1.03)	<0.01	1.03 (1.03–1.04)	<0.01
**Season at baseline**					
Summer	1717	Reference		Reference	
Fall	1422	1.12 (0.91–1.39)	0.29	0.88 (0.68–1.13)	0.31
Winter	4244	1.19 (1.00–1.42)	0.05	0.98 (0.79–1.20)	0.83
Spring	2636	0.99 (0.82–1.19)	0.91	0.84 (0.67–1.05)	0.12
**Season at follow up**					
Summer	1707	Reference		Reference	
Fall	1530	1.03 (0.87–1.22)	0.69	0.97 (0.78–1.20)	0.77
Winter	3554	0.99 (0.86–1.14)	0.95	0.96 (0.80–1.14)	0.64
Spring	3228	0.94 (0.81–1.09)	0.42	0.93 (0.77–1.12)	0.42
**Tobacco smoking status ^b^**					
Never smoker	2742	Reference		Reference	
Quit smoker	1402	1.33 (1.11–1.60)	<0.01	1.09 (0.86–1.37)	0.48
Current smoker	684	1.21 (0.95–1.54)	0.12	0.80 (0.61–1.07)	0.13
**Alcohol drinking status ^b^**					
Non-drinker	2569	Reference		Reference	
Drinker	4496	0.67 (0.59–0.76)	<0.01	0.77 (0.66–0.91)	<0.01
**Physical activity ^b^ at baseline**					
Low	2629	Reference		Reference	
Moderate	2004	0.60 (0.51–0.70)	<0.01	0.79 (0.65–0.97)	0.02
High	1917	0.35 (0.29–0.43)	<0.01	0.57 (0.45–0.71)	<0.01
**Physical activity change ^b^ during follow up**					
No improvement	2437	Reference		Reference	
Moderate improvement	1588	1.09 (0.93–1.28)	0.28	0.93 (0.75–1.14)	0.46
High improvement	920	0.90 (0.74–1.09)	0.29	0.82 (0.63–1.07)	0.14

25(OH)D, 25-hydroxyvitamin D; OR (95% CI), odds ratio with 95% confidence interval; # visits, numbers of follow up visits; nmol/L, nanomoles per liter. ^a^ The multivariate analysis are adjusted for all variables presented in the table. ^b^ Missing data was considered in the analysis as a separate category.

The estimates of the effect of temporal changes in serum 25(OH)D concentrations on metabolic syndrome at follow up are distinct when estimated through univariate *versus* multivariable analysis (Table 2) because this effect is strongly confounded by baseline serum 25(OH)D concentrations. Relative to visits without temporal increases in serum 25(OH)D concentrations, follow up visits with an increase of less than 25 nmol/L showed a 0.76 (95% CI: 0.62–0.93) time reduction in the risk for metabolic syndrome (Table 2). For visits with increases in serum 25(OH)D concentrations of 25 to less than 50 nmol/L, 50 to less than 75 nmol/L, and 75 nmol/L or more this was 0.64 (0.52–0.80), 0.59 (0.46–0.77), and 0.56 (0.44–0.70) respectively. Repeating this multivariable analysis while considering absolute changes in serum 25(OH)D concentrations rather than categories revealed a 6% reduction in the risk for metabolic syndrome (OR: 0.94; 95% CI: 0.90–0.97) for every increase of 25 nmol/L in serum 25(OH)D during follow up. This estimate did not change substantially when further adjusting for body mass index and HOMA-IR.

Metabolic syndrome at baseline strongly determined the syndrome at follow up (Table 2). Unlike smoking status, consumption of alcohol did affect the risk for metabolic syndrome in a statistically significant manner. However, changes in smoking and drinking status during follow up were small and did not allow for meaning analyses. Physical activity at baseline and increases in physical activity during follow up reduced the risk for metabolic syndrome at follow up.

The analyses presented in Table 3 confirm that higher 25(OH)D concentrations at baseline and larger increases in 25(OH)D during follow up reduce the risk of metabolic syndrome at follow up for the subgroup of participants without metabolic syndrome at baseline and for the subgroup of participants without metabolic syndrome at baseline and without reported use of vitamin D supplements prior to the baseline assessment.

**Table 3:** Risk for metabolic syndrome at follow up among participants without metabolic syndrome at baseline.

	Participants without Metabolic Syndrome at Baseline (*n* = 5510)			Participants without Metabolic Syndrome and Naïve to Vitamin D Supplementation at Baseline (*n* = 1930)		
	# visits	OR (95% CI) ^a^	*p*	# visits	OR (95% CI) ^a^	*p*
**Baseline 25(OHD), nmol/L**						
<50	1140	Reference		459	Reference	
50–<75	2144	0.78 (0.60–1.01)	0.06	910	0.83 (0.57–1.21)	0.32
75–<100	2159	0.49 (0.37–0.64)	<0.01	694	0.59 (0.38–0.91)	0.02
100–<125	1447	0.37 (0.27–0.52)	<0.01	287	0.57 (0.32–1.03)	0.06
≥125	1375	0.24 (0.16–0.34)	<0.01	205	0.17 (0.07–0.43)	<0.01
**Changes in 25(OH)D during follow up compared with baseline, nmol/L**						
No improvement	1680	Reference		421	Reference	
Increase of <25	2506	0.77 (0.60–0.98)	0.04	661	0.63 (0.39–1.00)	0.05
Increase of 25–<50	1644	0.62 (0.47–0.81)	<0.01	518	0.73 (0.46–1.17)	0.19
Increase of 50–<75	884	0.51 (0.37–0.71)	<0.01	339	0.56 (0.32–0.96)	0.03
Increase of ≥75	1551	0.60 (0.45–0.79)	<0.01	616	0.58 (0.36–0.93)	0.02

25(OH)D, 25-hydroxyvitamin D; OR (95% CI), odds ratio with 95% confidence interval; # visits, numbers of follow up visits; nmol/L, nanomoles per liter. ^a^ The analysis are adjusted for the variables included in the table, and additionally for gender, baseline age, season at baseline, season at follow up, tobacco smoking status, alcohol drinking status, and physical activity at baseline, physical activity change during follow up.

## 4. Discussion

Our analyses revealed that high serum 25(OH)D concentrations at baseline and increases in serum 25(OH)D concentrations during follow up independently contribute to a risk reduction of metabolic syndrome in a cohort of volunteer participants of a preventive health program who increased their average serum 25(OH)D concentrations from 89 nmol/L at baseline to 121 nmol/L during follow up.

Various cross-sectional studies revealed inverse associations of serum 25(OH)D and the presence of metabolic syndrome [14,15,16,17,18,19]. Prospective analyses of cohort studies revealed that baseline serum 25(OH)D concentrations determined the onset of metabolic syndrome. Kayeniyil *et al.* reported for an at risk population a 0.62 lower probability for onset of metabolic syndrome per standard deviation (23.3 nmol/L) increase in baseline serum 25(OH)D concentration over a three years follow up period [22]. For a population-based sample Gagnon *et al*. reported that participants in the lowest baseline 25(OH)D quintile (less than 45 nmol/L) had a 41% increased risk and those in the second quintile (45 to 57.5 nmol/L) a 74% increase risk for metabolic syndrome relative to those in the highest quintile (85 to 232.5 nmol/L) during the five years under study [21]. Skaaby *et al.* reported a 0.95 lower probability for onset of metabolic syndrome per 10 nmol/L increase in baseline 25(OH)D concentrations in a prospective population-based study followed for five years [23]. The above observations seem consistent with our observation of the effect on baseline serum 25(OH)D on metabolic syndrome. We acknowledge that comparisons across studies are not uncomplicated as studies vary in study objectives, follow up time, analytic approach, consideration of confounders, and baseline serum 25(OH)D concentrations. The average serum 25(OH)D concentration at baseline in our study (89 nmol/L) was higher than in the abovementioned cohort studies [21,23]. The baseline concentration was also higher than the 67.7 nmol/L reported for a nationally representative sample of Canadians [30]. This difference may result from self-selection whereby the program enrolled relatively more health aware volunteers seeking lifestyle counseling. This is reflected in fact that 49% of program participants reportedly used supplementation prior to enrollment whereas this is 34% for the general Canadian population [31]. We acknowledge that this self-selection may affect the generalizability of the findings but also note that the large heterogeneity in baseline 25(OH)D concentrations improved our ability to examine the effect of baseline concentration for metabolic syndrome. In this respect, we observed an effect of baseline 25(OH)D on metabolic syndrome that was by and large maintained after adjustment for body mass index and HOMA-IR at baseline, whereas this was not consistently the case in the above referred to cohort studies [21,22,23]. The observation that vitamin D status predicts the incidence of metabolic syndrome independently of body mass index and HOMA-IR is important to the recognition of vitamin D status as a causal factor in the etiology of the syndrome. Our other observation that increases in serum 25(OH)D during follow up reduce the risk for metabolic syndrome also supports the hypothesis that vitamin D status is a causal factor.

Al-Daghri *et al.* [24] reported on an intervention program to promoted responsible sun exposure and increased intake of vitamin D rich foods. The 69 overweight and obese participants of this program experienced an increase in serum 25(OH)D concentration from 18.7 nmol/L at baseline to 30.7 nmol/L after one year and a parallel drop in metabolic syndrome prevalence for 24.6% to 13.0%. This program would not work in Canada, because of the Northern latitude and limited cutaneous vitamin D synthesis. Canadian diets only contribute an estimated 232 IU of vitamin D per day, and thus supplementation seems essential [32]. The study by Al-Daghri *et al.* and ours both studied increases in 25(OH)D concentrations though at the outmost ranges. Al-Daghri *et al.* reported average change from 18.7 nmol/L at baseline to 30.7 nmol/L at one year, whereas we observed and increase from 89 nmol/L at baseline to 121 nmol/L at the follow up visit. The fact that both studies have generated positive findings is encouraging. As neither study was a randomized controlled trial, we recommend the latter to further establish the potential of vitamin D in the prevention of metabolic syndrome.

Findings of the present study suggested that promotion of adequate vitamin D status may reduce the public health burden of metabolic syndrome and potentially that of type 2 diabetes and cardiovascular disease as studies have shown that metabolic syndrome increases the risk for type 2 diabetes six fold and the risk for cardiovascular disease two fold [3,4]. The prevalence of type 2 diabetes is on the rise and across all age groups among Canadians [33]. Strategies to curb this trend are in demand, not only from a clinical perspective but also from an economic perspective. In the province of Alberta, Canada, with a population of about four million, total health-care cost for the management of diabetes was estimated to be about 700 million Canadian dollars in 2008, and projected to reach two billions Canadian dollars in 2035 [34]. If we assume the 6% reduction in the prevalence of metabolic syndrome revealed in the present study, would translate into a 6% reduction in diabetes, promotion of vitamin D that achieves a 25 nmol/L increase in serum 25(OH)D may reduce health care costs for the management of diabetes with approximately 67 million Canadian dollars in 2015 (that is 6% of the estimated costs of 1121 million dollars in 2015) [34].

The longitudinal design, the large sample size and the wide range of serum 25(OH)D concentrations are strengths of the present study. Limitations pertain to the fact that this was not a randomized controlled trial and thus the results may be biased. We therefore recommend the present findings be tested in trials with blinding and random allocation. Another limitation may pertain to the fact that participants may have adjusted their lifestyles after enrollment, and their metabolic syndrome status may have changed as a result. The potential bias resulting from these influences will be isolated in a trial with blinding and random allocation as suggested above.

## 5. Conclusions

The present study revealed that improvements of vitamin D status may reduce the prevalence of metabolic syndrome. Herewith the study suggests that vitamin D supplementation may further reduce the public health burden for metabolic syndrome, and possible subsequent health conditions including type 2 diabetes and cardiovascular disease.
